# Progression of Post-Traumatic Osteoarthritis in rat meniscectomy models: Comprehensive monitoring using MRI

**DOI:** 10.1038/s41598-018-25186-1

**Published:** 2018-05-01

**Authors:** Tonima S. Ali, Indira Prasadam, Yin Xiao, Konstantin I. Momot

**Affiliations:** 1 Queensland University of Technology (QUT), Brisbane, Queensland (QLD) Australia; 2 Institute of Health and Biomedical Innovation, Kelvin Grove, QLD 4059 Australia

## Abstract

Knee injury often triggers post-traumatic osteoarthritis (PTOA) that affects articular cartilage (AC), subchondral bone, meniscus and the synovial membrane. The available treatments for PTOA are largely ineffective due to late diagnosis past the “treatment window”. This study aimed to develop a detailed understanding of the time line of the progression of PTOA in murine models through longitudinal observation of the femorotibial joint from the onset of the disease to the advanced stage. Quantitative magnetic resonance microimaging (µMRI) and histology were used to evaluate PTOA-associated changes in the knee joints of rats subjected to knee meniscectomy. Systematic longitudinal changes in the articular cartilage thickness, cartilage *T*_2_ and the *T*_2_ of epiphysis within medial condyles of the tibia were all found to be associated with the development of PTOA in the animals. The following pathogenesis cascade was found to precede advanced PTOA: meniscal injury → AC swelling → subchondral bone remodelling → proteoglycan depletion → free water influx → cartilage erosion. Importantly, the imaging protocol used was entirely MRI-based. This protocol is potentially suitable for whole-knee longitudinal, non-invasive assessment of the development of OA. The results of this work will inform the improvement of the imaging methods for early diagnosis of PTOA.

## Introduction

A common consequence of joint injury is post-traumatic osteoarthritis (PTOA), which accounts for 12% of all cases of osteoarthritis (OA)^1^. Trauma sustained by joint tissues, particularly tears of the meniscus or anterior cruciate ligament (ACL) can result in injuries to articular cartilage (AC) and lead to the development of PTOA within a 10-to-15 years’ time window. Characterised primarily by gradual degradation of articular cartilage (AC)^2^, the pathogenesis of PTOA also includes bone remodelling^3–7^, meniscal modification^8^ and synovial inflammation^9,10^. However, PTOA is often detected in advanced stage after it becomes radiographically apparent by the altered alignment of the major bones caused by severe cartilage damage. The cartilage is translucent to the X-ray radiography used in clinical practice and therefore is not capable of detecting the cartilage degradation at the onset of PTOA. The discordance between radiological and clinical OA findings^11^ also highlights major limitations of the conventional radiography. With no cure available until now, preventative measures or clinical intervention within the ‘treatment window’ of early PTOA may provide the optimal clinical outcome for disease management^12^.

PTOA cases are commonly reported by patients as a result of pain in the joints followed by structural damage and functional impairment at the advanced stage^13–15^, which makes it difficult to investigate the early PTOA in human patients. With an incomplete understanding of early PTOA, the sequence of events leading towards symptomatic PTOA remains unclear as well. In the present study, we investigated the progression of PTOA in rat knee joints that underwent meniscectomy (MSX), a standard protocol for PTOA initiation known to replicate human PTOA with a significant degree of similarity^16,17^. The use of this model has also eliminated the variabilities due to age, weight, genetics, and environmental conditions that may result in a significant variability of the clinical manifestation of the disease. The whole knee joints were monitored weekly over an eight-week post-injury time window in order to capture the gradual developmental changes from very early PTOA preceding to the severe stage.

Magnetic Resonance Imaging (MRI), with its recent advancements, is now extremely sensitive to the changes in cartilage^18–21^, subchondral bone marrow^4^ and synovial membrane^22,23^. The transverse spin relaxation time constant (*T*_2_) of MRI is sensitive to the state of the water in biological tissues and also to the 3D architecture of the collagen scaffold within the cartilage extracellular matrix (ECM)^24–27^. Consequently, *T*_2_ mapping allows an indirect assessment of the integrity and the microscopic organisation of the ECM of cartilage^28–33^, which generally correlate with cartilage damage observed in model PTOA^34,35^. The resolution and signal to noise ratio (SNR) of MRI can be enhanced further by using stronger magnetic fields in the micro-MRI (µMRI) system. With the advantage of 3D imaging capability of µMRI, we have examined all tissues of the whole knee joints of our rat PTOA model using µMRI and quantitative *T*_2_ relaxation mapping. In our analysis, we have further emphasised on the tibial hyaline cartilage and tibial tissues, which remain less studied by MRI mostly due to their irregular shapes and the difficulty in isolating them from adjacent structures. The µMRI results were compared against histology assays in order to confirm that the joints that underwent MSX reached severe PTOA within the 8-week observational time period.

To date, numerous studies have investigated the tissues of the knee joint both in normal and in PTOA-affected states^2–9,17,36–39^. Nevertheless, the tissue alteration pathway leading towards symptomatic PTOA has not yet been identified due to late diagnosis and analytical limitations. The objectives of the present study were (1) to enhance the analytical capabilities of quantitative MRI for early detection of PTOA and (2) to identify the sequential changes in tibial tissues leading from the initial knee injury towards advanced PTOA.

## Results

**Figure 1:**
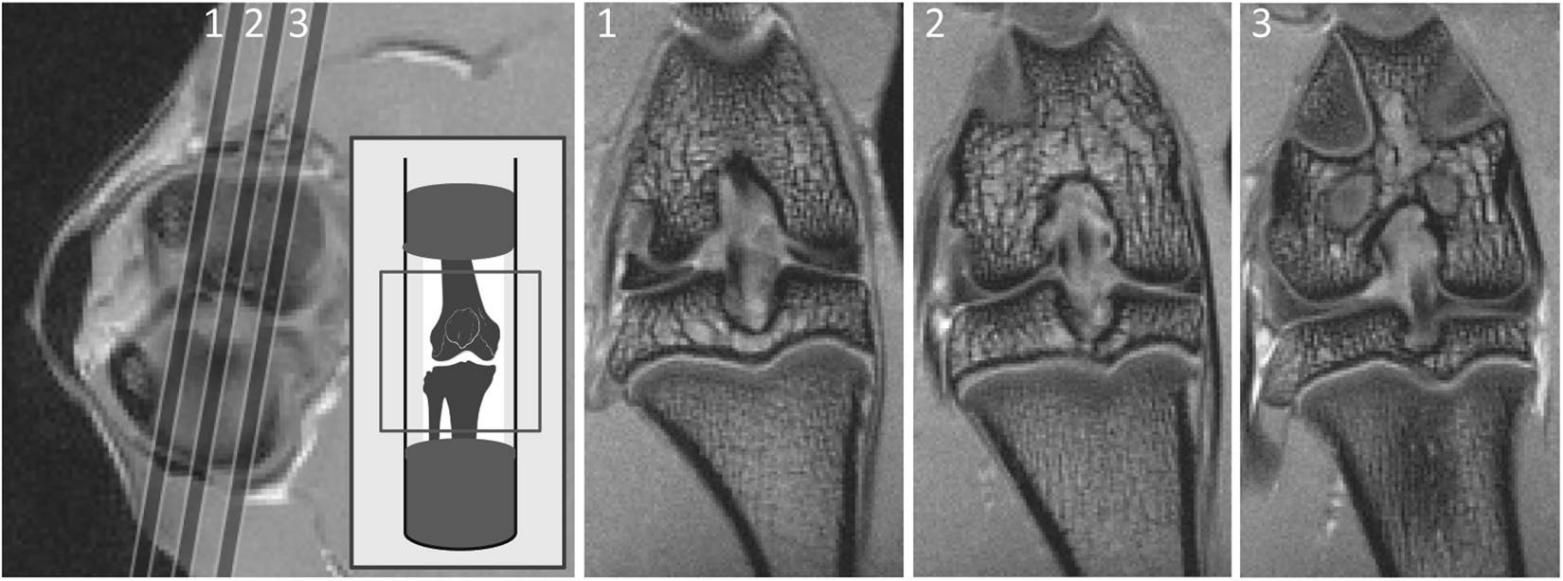
The MRI scan locations are shown in an axial slice of control knee joint. The position of coronal slice inside MRI gantry is shown in inset. Here 1, 2 and 3 refer to the anterior, central and posterior slices, respectively. These slice orientations were maintained for all scans of CTRL, MSX and CLAT joints. The femoral and tibial AC, the menisci, cortical and trabecular bone of the epiphysis, ligaments and fat tissues were clearly visible in the *T*_2_-weighted coronal slices acquired maintaining this protocol. The schematic outline of the knee in the inset is reproduced from https://en.wikipedia.org/wiki/Knee#/media/File:Knee_skeleton_lateral_anterior_views.svg in accordance with the terms of the CC BY 2.5 license.

Figure 1 shows the location and the orientation of the three coronal MRI slices acquired from a control (CTRL) joint at week 1. All of the knee joints that were investigated in this study were imaged with the same sample position and slice orientation. The femoral and tibial AC were in direct contact in the central (second) slice, which also contained the largest cross-section of ACL and posterior cruciate ligament (PCL). In this slice location, osteophyte-like growths were observed at the medial tibial condyles of the joints that underwent meniscectomy (MSX joints) at every weekly time point between week 4 and week 8. In contrast, the lateral condyles did not exhibit osteophytes-like growths or any significant changes in the AC or subchondral bone over the study period (according to Mann-Kendall trend test).

### Histological Analysis

**Figure 2:**
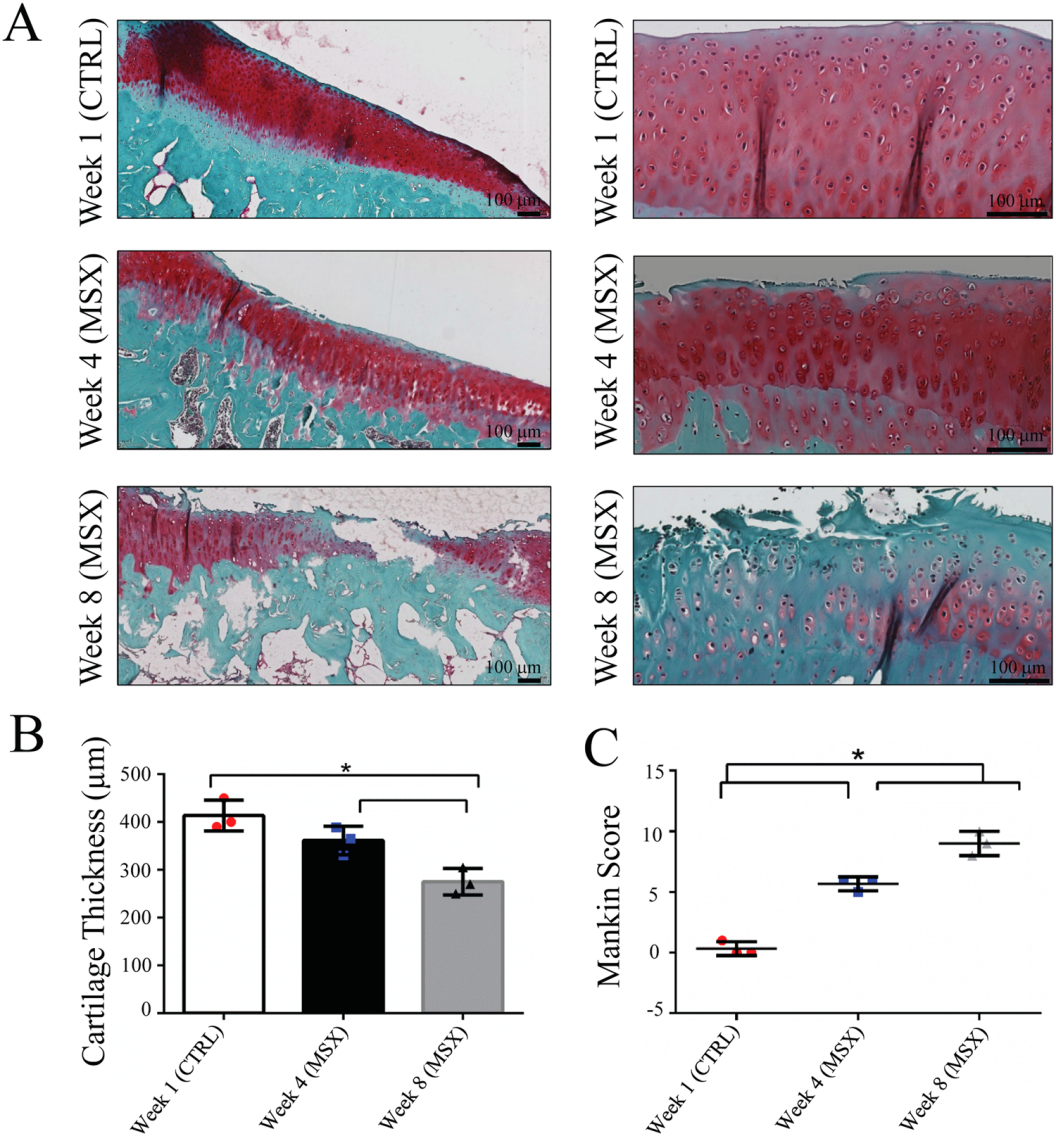
Cartilage sections of medial condyles of CTRL and MSX joints (**A**) stained with safranin-O fast green, which provided colour discrimination between bone and cartilage. Here, the cartilage matrix proteoglycan is stained red, cell nuclei black, cytoplasm grey green, and the underlying bone green^73^. Week 1 (CTRL) showed abundant proteoglycan, week 4 (MSX) showed proteoglycan depletion while week 8 (MSX) showed major proteoglycan loss. Gradual thinning of cartilage was observed at week 4 and week 8 as shown in (**B**). The Mankin scores of these slices are plotted in (**C**).

Three histology slices of the AC are shown in Fig. 2. These were sectioned from the medial tibial compartment of a CTRL joint at week 1, a MSX joint at week 4, and a MSX joint at week 8. Cartilage surface roughness, fibrillation, small osteophytes and areas with peripheral fibrous tissue proliferation were observed in the week 4 and week 8 MSX samples. The proteoglycan content was lower in the week 8 MSX sample than in the week 4 MSX sample, which in turn was lower than in the CTRL sample. The same pattern was observed for the AC thickness of the three samples (Fig. 2B). The Mankin Scores^40^ of these samples (Fig. 2C) validate the presence of PTOA in the MSX joints and confirm that the disease had advanced in severity from week 4 to week 8. Mankin score takes account of PG depletion and cell count in cartilage and is considered to be the standard procedure for evaluating Osteoarthritis^41^.

### Thickness and *T*_2_ of Articular Cartilage: Temporal Evolution and Mutual Correlation

**Figure 3:**
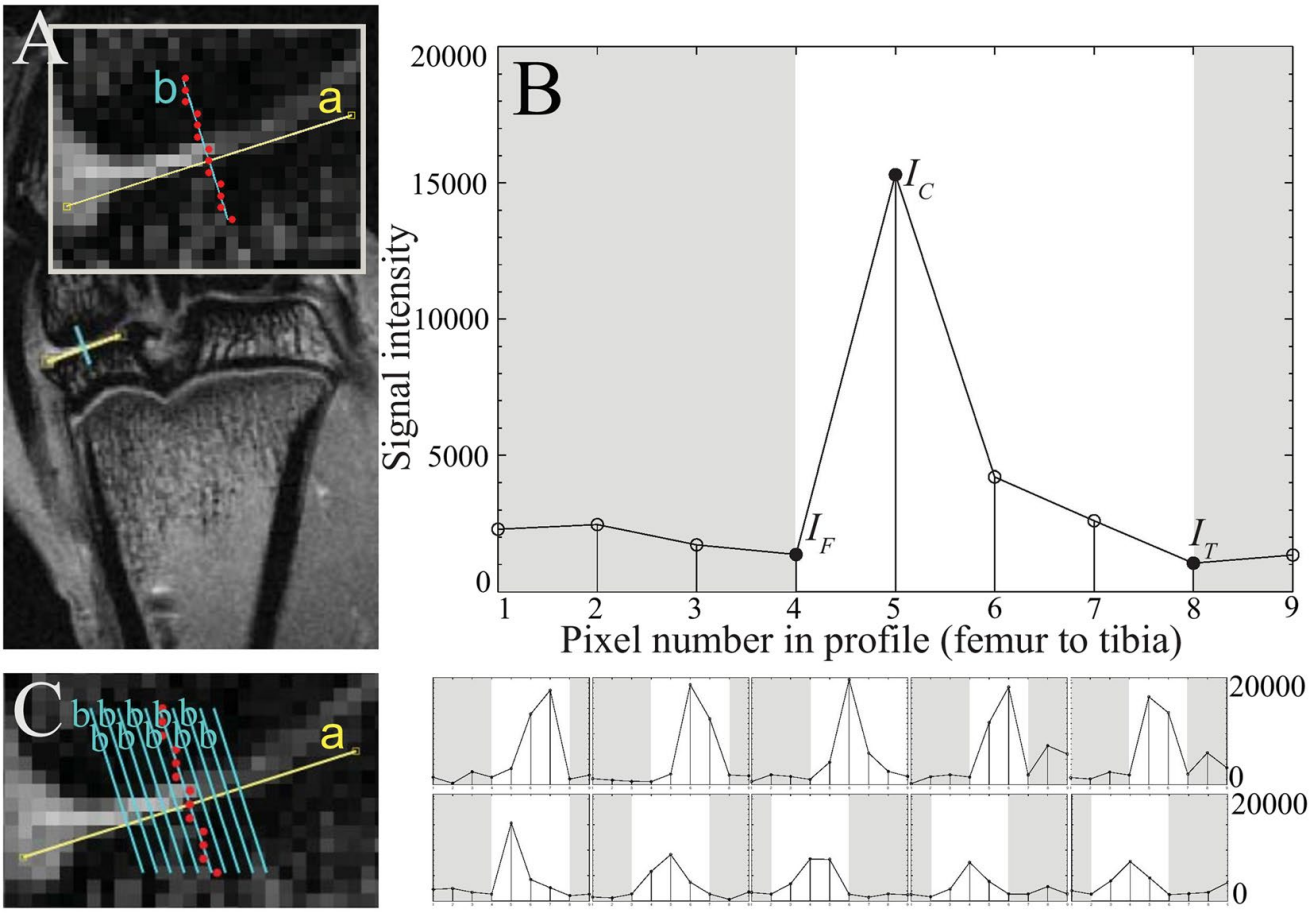
The cartilage thickness measurement procedure shown in a *T*_2_ weighted MR image of a MSX joint at TE = 12 ms (**A**). The straight line bordering AC is shown in yellow and denoted by a. The perpendicular line drawn from femur to tibia, b, is shown in blue in the inset, the nearest voxels of line b are shown in red. The corresponding *T*_2_ profile in (**B**) represents femoral cortical bone in pixel 1–4, cartilage in pixel 5, partial volume of cartilage in pixel 6–8 and tibial cortical bone in pixel 8–9. The partial volume effect observed in pixels 6–8 was corrected by using Eqs (2) and (3). Cartilage thickness was computed by multiplying the total number of voxels representing cartilage with voxel resolution (78 µm). All of the perpendicular lines b and corresponding *T*_2_ profiles are shown in (**C**). The partial volumes of each profile was corrected as above and a thickness was computed. The mean cartilage thickness was computed by averaging the thicknesses of these intensity profiles.

**Figure 4:**
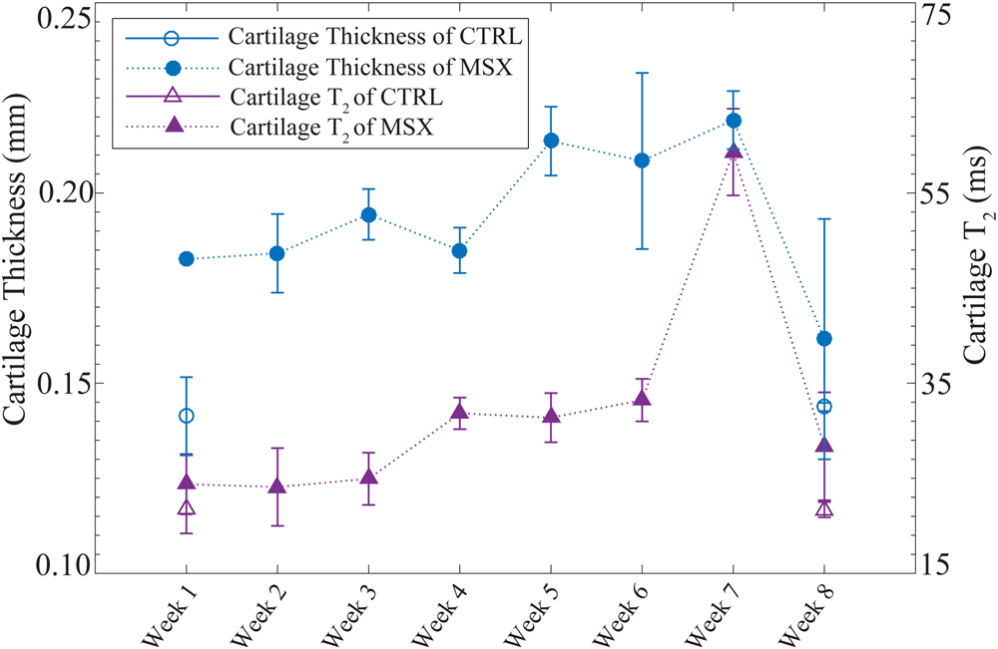
Cartilage thickness and cartilage *T*_2_ evolution of MSX joints over the eight week observation period post meniscectomy. The CTRL data of week 1 and week 8 are also presented here. Cartilage *T*_2_ exhibited little change between week 1 and week 3, as well as between week 4 and week 6. The data represent cartilage from the medial condyle of central coronal slice. Data plotted as mean ± SE.

With partial volume correction, as shown in Fig. 3 for a MSX joint, the thickness of AC in medial tibial condyle varied between 2 and 4 pixels. Figure 4 shows the temporal evolution of the AC thickness and the AC *T*_2_ for the medial tibial condyle of the MSX joints, both measured on the central coronal slice. The cartilage thickness showed a tendency to increase for the first 7 weeks with a prominent increment at week 5 and a substantial drop after week 7. A significant monotonic trend (Mann-Kendall trend test, *p* < 0.02) was observed for the week 1–week 7 time period. No net trend was observed when week-8 data was included. The AC *T*_2_ also showed a tendency to increase for the first 7 weeks following the surgery with a strong monotonic trend (Mann-Kendall trend test, *p* < 0.02). The *T*_2_ increased rapidly between week 6 and week 7 followed by a quick drop between week 7 and week 8. During the 8–week-long observation period after meniscectomy, both the highest AC thickness and the longest AC *T*_2_ were observed at week 7. Additionally, the thickness of AC and its *T*_2_ were found to have strong correlation (Spearman’s rank order correlation, *p* < 0.05) throughout these eight weeks.

### Temporal Evolution of the Epiphyseal *T*_2_

**Figure 5:**
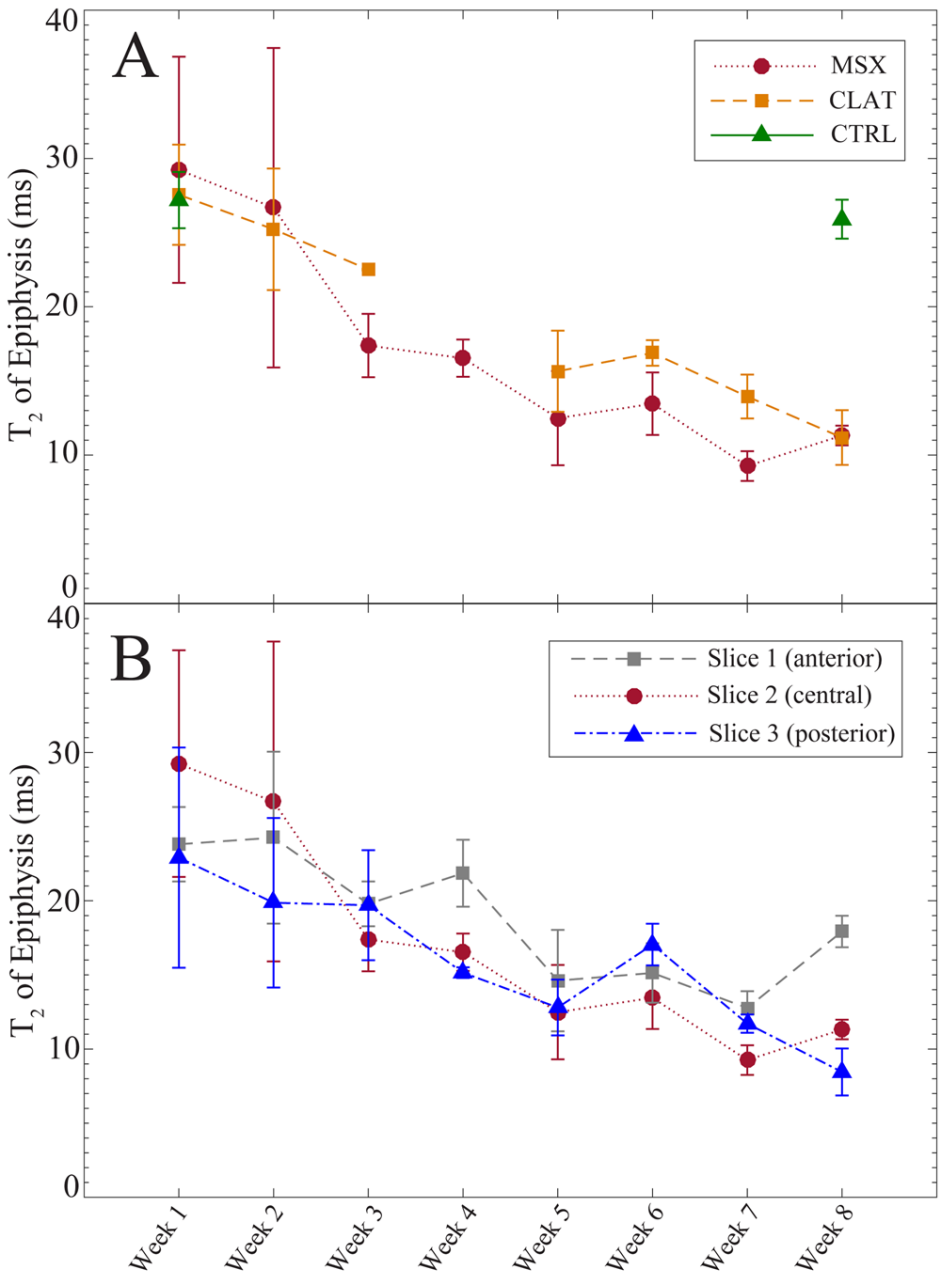
Mean *T*_2_ of medial epiphysis of CTRL, MSX and CLAT joints over the eight week observation period post meniscectomy (**A**). The data represent epiphyseal *T*_2_ measured from central coronal slice location. The epiphyseal *T*_2_ of medial condyles of anterior (slice 1), central (slice 2) and posterior slice (slice 3) locations for the MSX joints are shown in (**B**). Data plotted as mean ± SE.

The average *T*_2_ of medial epiphysis, which included both the cortical/subchondral bone and the trabecular bone within the epiphysis, exhibited gradual changes over the 8-week observation period in both MSX and contralateral (CLAT) joints. Figure 5A shows the evolution of epiphyseal *T*_2_ in the medial tibial condyle (the central coronal slice) for the CTRL, MSX and CLAT joints. The CTRL joints showed no significant changes between week 1 and week 8, the epiphyseal *T*_2_ remained over 25 ms at both time points. For the MSX joints, the epiphyseal *T*_2_ continually decreased for seven weeks after the surgery and reached 9.2 ms at week 7 (See Table 1). This was followed by a slight increase at the week 8 time point. This temporal evolution of epiphyseal *T*_2_ was associated with a very strong monotonic trend (Mann-Kendall trend test, *p* < 0.01) for the week 1–week 8 time period. By visual observation, thinning of the subchondral bone was identified in the first two weeks following surgery, which was followed by gradual thickening of the subchondral bone from week 3 to week 8.

**Table 1:** .

Cartilage Thickness (µm)								
	Week 1	Week 2	Week 3	Week 4	Week 5	Week 6	Week 7	Week 8
CTRL	141 ± 10	—	—	—	—	—	—	144 ± 1
MSX	182 ± 0	184 ± 10	194 ± 7	185 ± 6	214 ± 9	208 ± 23	219 ± 8	161 ± 31
CLAT	179 ± 3	161 ± 12	129 ± 7	—	128 ± 6	130 ± 15	139 ± 9	162 ± 16
**Cartilage T**_**2**_ **(ms)**								
CTRL	21.7 ± 2.5	—	—	—	—	—	—	21.7 ± 1.4
MSX	24.4 ± 3.2	24.1 ± 4.1	24.9 ± 2.7	31.8 ± 1.7	31.4 ± 2.6	33.2 ± 2.2	59.3 ± 4.6	28.4 ± 5.7
CLAT	22.5 ± 1.5	21.2 ± 2.2	18.2 ± 1.5	—	22.3 ± 2.8	19.0 ± 1.1	21.8 ± 1.9	17.6 ± 4.4
**T**_**2**_ **of Epiphysis (ms)**								
CTRL	27.2 ± 1.9	—	—	—	—	—	—	25.9 ± 3.3
MSX	29.2 ± 7.6	26.7 ± 10.8	17.4 ± 2.1	16.5 ± 1.2	12.5 ± 3.2	13.4 ± 2.1	9.2 ± 1.0	11.3 ± 0.7
CLAT	27.5 ± 3.4	25.2 ± 4.1	22.5 ± 0.1	—	15.6 ± 2.7	16.9 ± 0.9	13.9 ± 1.5	11.2 ± 1.8

The thickness of AC, its *T*_2_ and the *T*_2_ of epiphysis of the medial tibial condyle for the CTRL, MSX and CLAT groups for each observation week. All of these physical quantities are measured from the *T*_2_-weighted MR images and *T*_2_ maps of the central coronal slice location shown in Fig. 1. Data is presented as mean ± standard error.

In the CLAT joints, the epiphyseal *T*_2_ was observed to continually decrease for the eight weeks after surgery. The epiphyseal *T*_2_ was 11.2 ms by week 8. A very strong monotonic trend was identified for the epiphyseal *T*_2_ of CLAT joints (Mann-Kendall trend test, *p* < 0.01) in the week 1–week 8 time period. Visual observation confirmed initial thinning of subchondral bone (week 1 to week 3) that was followed by gradual thickening (week 4 to week 8) within the study period. The epiphyseal *T*_2_ in the MSX and CLAT joints were found to be statistically correlated with each other (Spearman’s rank order correlation analysis, *p* < 0.01).

Figure 5B illustrates the spatial variations in the epiphyseal *T*_2_ of medial tibia for the three coronal slice locations of MSX joints. The epiphyseal *T*_2_ exhibited similar patterns in progression in all three slices with a gradual decrease for eight weeks. A slight increase at week 6 time point was also observed in all three slices. The epiphyseal *T*_2_ of the anterior slice and the central slice showed a slight increase at week 8, reaching 17.9 ms and 11.3 ms, respectively, by week 8. However, in the posterior slice, the epiphyseal *T*_2_ continually decreased for eight weeks and reached 8.4 ms at week 8. The epiphyseal *T*_2_ in the anterior, central and posterior slices were found to be statistically correlated according to Spearman’s rank order correlation analysis (anterior and central slice: *p* < 0.01, posterior and central slice: *p* < 0.01, anterior and posterior slice: *p* < 0.05). Initial thinning and gradual thickening of subchondral bone was identified in all three slice locations by visual observation.

### Whole-Joint Evaluation of the Longitudinal Changes

**Figure 6:**
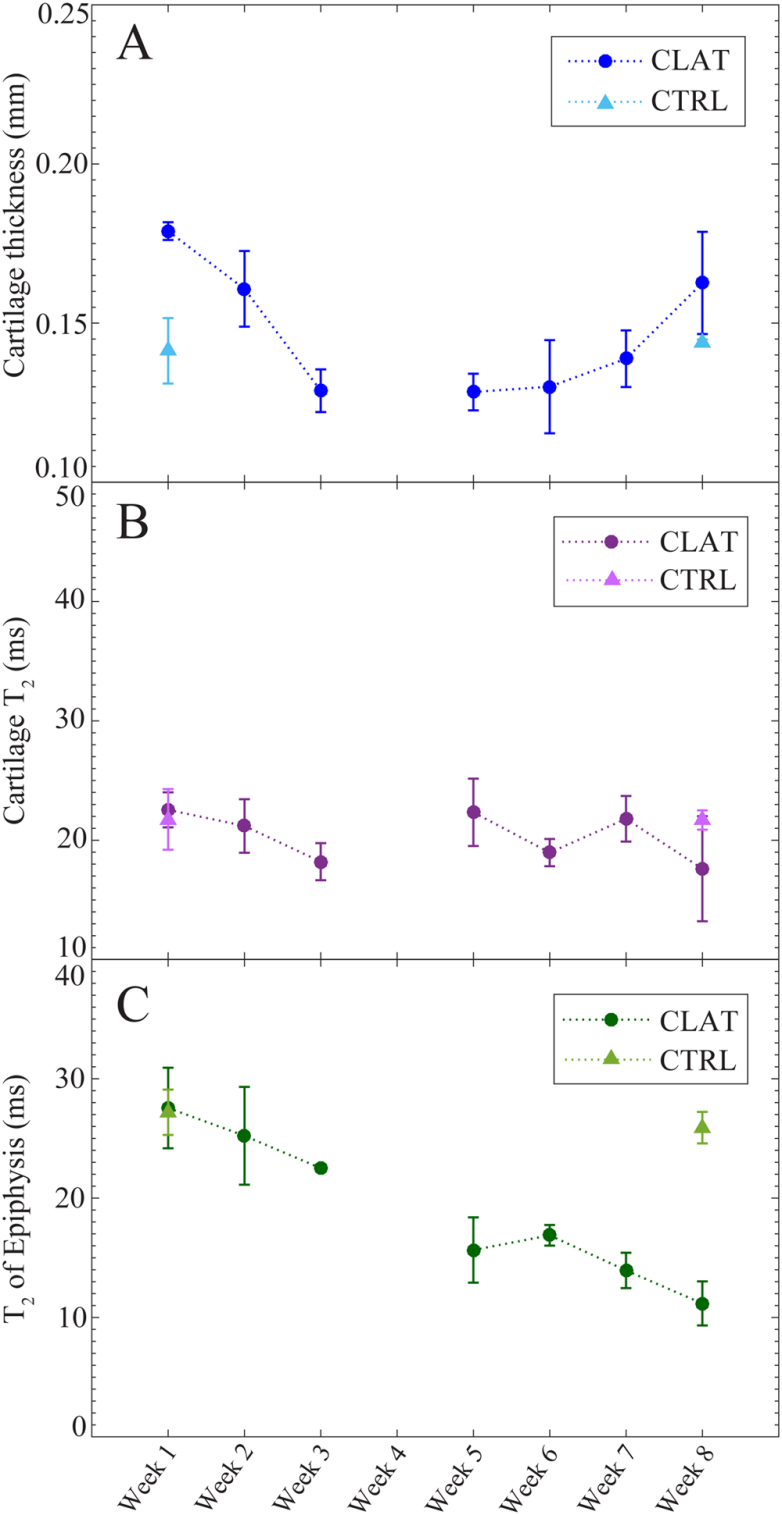
Changes in the tissues of the medial condyles of CLAT joints, in comparison to controls, during the eight week observation period post meniscectomy. Cartilage thickness (**A**), cartilage *T*_2_ (**B**) and *T*_2_ of epiphysis (**C**) of CTRL and CLAT joints are plotted for week 1–week 8 for central coronal slice location. Data plotted as mean ± SE.

**Figure 7:**
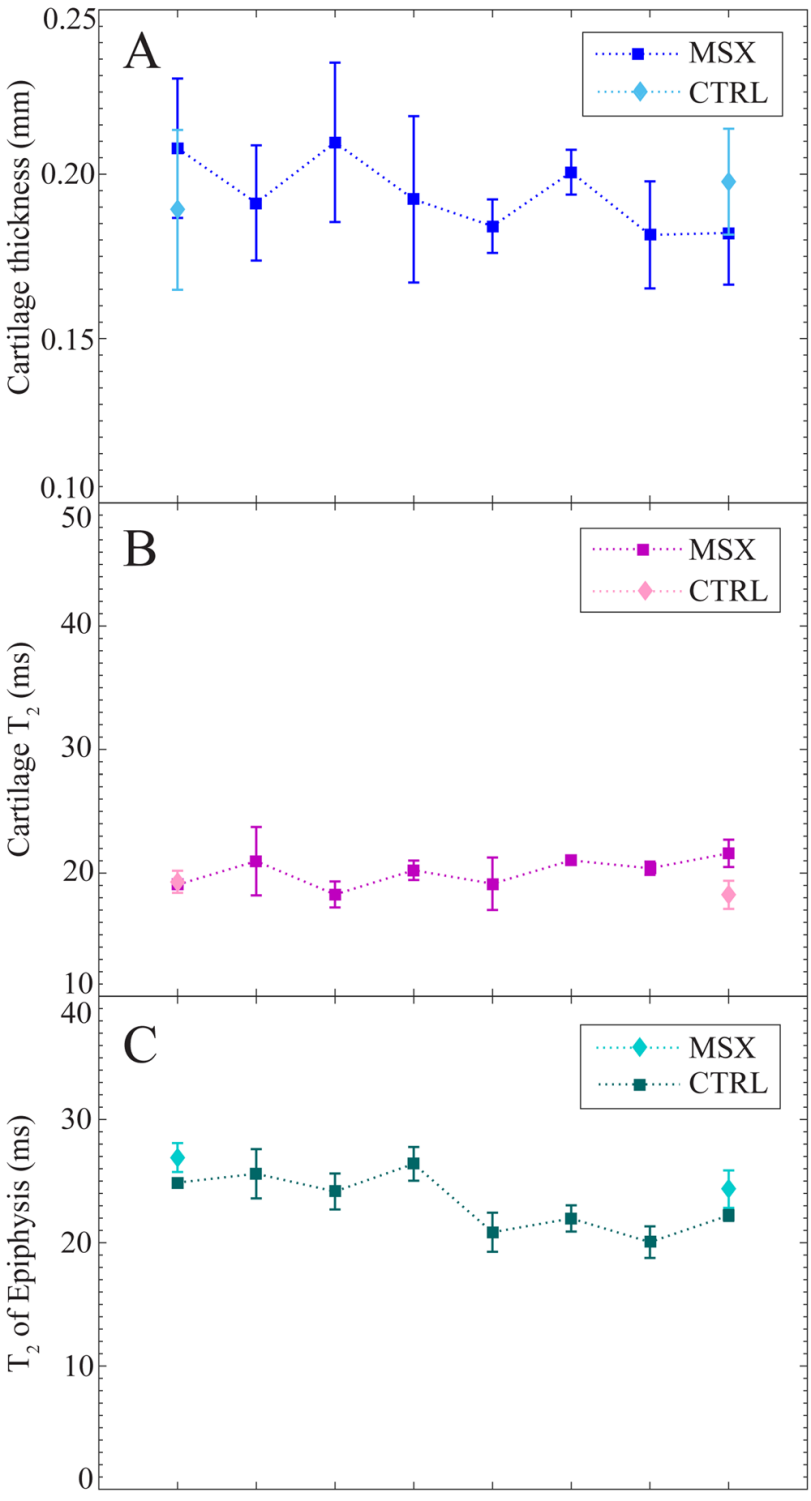
Changes in the tissues of the lateral condyles of MSX joints, in comparison to lateral condyle controls, during the eight week observation period post meniscectomy. Cartilage thickness (**A**), cartilage *T*_2_ (B) and *T*_2_ of epiphysis (**C**) of CTRL and MSX joints are plotted for week 1–week 8 for central coronal slice location. Data plotted as mean ± SE.

AC thickness, AC *T*_2_ and epiphyseal *T*_2_ were measured throughout the observation period, from week 1 to week 8 for CTRL, MSX and CLAT joints. These quantities are plotted in chronological orders in Fig. 6 for the medial condyles of CLAT joints. Figure 7 exhibits the same quantities measured from the lateral condyles of the MSX joints. The CTRL data of each quantity was also plotted in both Figs 6 and 7 in order to demonstrate the changes only due to the joint maturation process from week 1 to week 8.

Table 1 reports the thickness of AC, its *T*_2_ and the *T*_2_ of epiphysis for the CTRL, MSX and CLAT joints for each observation week. Table 2 presents the intragroup changes in AC thickness, its *T*_2_ and the *T*_2_ of epiphysis of the medial tibial condyle from week 1 to week 7 and from week 1 to week 8 for CTRL, MSX and CLAT joints.

**Table 2:** The intragroup changes in AC thickness, AC *T*_2_ and *T*_2_ of epiphysis of the medial tibial condyle, for CTRL, MSX and CLAT joints.

			Slice 1 (anterior)	Slice 2 (middle)	Slice 3 (posterior)
Change in Thickness of Articular Cartilage (µm)	CTRL	Δ_8−1_	−15 ± 5	2 ± 10	−3 ± 10
	MSX	Δ_7−1_	54 ± 19	36 ± 8	3 ± 46
		Δ_8−1_	−30 ± 26	−21 ± 31	−33 ± 22
	CLAT	Δ_7−1_	12 ± 13	−40 ± 9	47 ± 11
		Δ_8−1_	3 ± 12	−16 ± 16	27 ± 6
Change in T_2_ of Articular Cartilage (ms)	CTRL	Δ_8−1_	0.8 ± 3.1	−0.04 ± 2.6	2.4 ± 3.3
	MSX	Δ_7−1_	19.4 ± 3.4	34.9 ± 5.6	20.6 ± 2.6
		Δ_8−1_	24.1 ± 5.3	4.0 ± 6.4	40.5 ± 2.8
	CLAT	Δ_7−1_	−2.3 ± 0.7	−0.7 ± 2.4	−7.8 ± 1.6
		Δ_8−1_	−3.3 ± 2.2	−4.9 ± 4.6	−5.8 ± 2.2
Change in T_2_ of Epiphysis (ms)	CTRL	Δ_8−1_	−4.3 ± 2.0	−1.3 ± 2.7	−3.7 ± 1.7
	MSX	Δ_7−1_	−11.4 ± 2.7	−20.0 ± 7.7	−11.2 ± 7.4
		Δ_8−1_	−5.9 ± 2.7	−17.9 ± 7.6	−14.4 ± 7.6
	CLAT	Δ_7−1_	−7.1 ± 4.1	−13.6 ± 3.7	−7.2 ± 2.1
		Δ_8−1_	−7.8 ± 4.1	−16.4 ± 3.8	−10.2 ± 1.3

Δ_7−1_ is defined as the difference between week 7 and week 1 for the given physical quantity (*A*), the given slice location, and the given group: Δ_7−1_ = *A*_7_ (group, slice) − *A*_1_ (group, slice). Equivalent definition was used for Δ_8−1_: Δ_8−1_ = *A*_8_ (group, slice) − *A*_1_ (group, slice). The respective quantities were measured from *T*_2_-weighted MR images and *T*_2_ maps at the three different coronal slice locations shown in Fig. 1. Data is presented as mean ± standard error.

## Discussion

This study investigated the development of PTOA in a rat model where the disease was induced by complete removal of medial meniscus or meniscectomy^16^. The menisci of the knee joint protect the ends of the bones from rubbing against each other and provide shock absorption and load transmission^42,43^. Meniscectomy, either partial or total, disturbs the natural loading mechanism of a knee joint, which in turn increases the amount of strain on the AC. The absence of a meniscus in the knee joint has been linked to “over-compression” of the cartilage as well as a slower post-load recovery. These effects, in turn, have been postulated to set off a cascade of cellular and structural events leading to the development of OA^3,44,45^. In our rat meniscectomy model, the AC layers covering the medial condyles, both in tibia and in femur, were exposed to an increased risk of ECM degradation or OA initiation. We examined all tissues of the MSX and CLAT joints and compared the respective tissues against that of the age matched controls using µMRI. By this, three physical quantities were identified that consistently evolved with the progression of PTOA: the thickness of AC, *T*_2_ of AC, and *T*_2_ of epiphysis. The use of age-matched controls ensured that there was no bias inherent to the maturation process of the rat joints.

The imaging pulse sequence used in this study, Multi-Slice Multi-Echo (MSME) imaging, is not the most common imaging sequence for anatomical visualisation of articular cartilage; fast gradient echo-based pulse sequences such as FLASH are more commonly used for this purpose^30^. Nevertheless, MSME was used in our study because one of its key objectives was to obtain quantitative *T*_2_ maps of the joints, and MSME allowed obtaining these in a time-efficient manner.

### Swelling and Degradation of Articular Cartilage

The partial volume correction by Eqs (2) and (3) allowed the determination of AC thicknesses that were not integer multiples of the voxel size (78 µm), which effectively improved the precision of thickness measurement in MRI. The thickening of AC was observed as early as week 1 in the MSX joints, which continued to thicken for seven consecutive weeks with a strong monotonic trend (Mann-Kendall trend test, *p* < 0.02). The rate of change in the MSX joints significantly exceeded that in the CTRL joints (see Table 1). By comparing these results with the results of histological analysis, it was concluded that weeks 1–7 corresponded to the gradual depletion of proteoglycan and cellular loss, which in turn allowed the AC to swell. At week 8, a severe loss of AC thickness was observed in the *T*_2_-weighted image, with the average week-8 thickness being lower than that at week 1. Histological results indicated that week 8 corresponded to the erosion of AC (Fig. 2A). It should be noted that the histology-based thickness measurements were performed on dehydrated sections, which did not reflect the swelling of AC present in the native samples. Therefore, the AC thickness in the histological samples cannot be taken as an indicator of the AC thickness in the actual intact knee.

Cartilage thickening or swelling in early OA have been observed by MRI in previous studies^18–20^. A study of a spontaneous model of OA in guinea-pigs reported an initial increase of AC thickness for 24 weeks followed by a decrease for the next 28 weeks^20^. In a rabbit partial meniscectomy model of PTOA, the cartilage thickness at the weight-bearing area of the femoral medial condyle increased for eight weeks following surgery and then decreased at week 10^18,19^. However, the thickness of tibial cartilage showed a significant increase only at week 6^18^. Our study, along with those cited above, supports the model whereby the AC thickens following the removal of meniscus, and continues to thicken until it reaches a maximum thickness supported by its ECM. After this time point, AC loses its thickness due to erosion of the ECM. The complete medial meniscectomy employed in our model can be assumed to have more severe effects on weight bearing than partial meniscectomy. Therefore, it can be expected to result in a more aggressive progression of PTOA comparing to the above mentioned PTOA models, which is in agreement with our observations.

### Evolution of *T*_2_ in Articular Cartilage

The transverse spin relaxation time constant (*T*_2_) is a valuable indicator of the water content in AC. The water content, in turn, is strongly related to the integrity of the proteoglycan-collagen matrix^28,29^. The reduced proteoglycan content in PTOA disrupts the collagen network in AC^46^, which increases its permeability to water; this results in an elevated water content and longer *T*_2_ values^47^. Literature suggests that the modified *T*_2_ of cartilage could be used as a harbinger of the onset of PTOA before the disease reaches its radiographic stage^48^. Our weekly observations of quantitative *T*_2_ maps confirmed the presence of *T*_2_ changes in the AC of the MSX joints from week 1 to week 8.

The spin relaxation rate constants, 1/*T*_2_, of AC can be viewed as the weighted average of the contributions from two components: bound water (BW), which is transiently associated with the ECM biomacromolecules (collagen and proteoglycans) and has a short intrinsic *T*_2_, and free water (FW), which experiences a molecular environment similar to that of bulk water and has a long intrinsic *T*_2_^21,25,49,50^. The *T*_2_ values therefore exhibit an inverse relationship with the local concentration of proteoglycans^47^. However, cartilage *T*_2_ is also influenced by the *T*_2_ magic-angle effect^25,51,52^, which is due to the aligned collagen fibers anisotropically restricting the rotational dynamics of bound water^53,54^. The collagen orientation varies throughout the AC, which typically divides the AC into three histological zones: superficial, transitional and radial that differ in *T*_2_ values^36,55^. The AC observed in this study was rather thin and it was not possible to differentiate one zone from another. For every sample, therefore, an average *T*_2_ value was computed from AC that combined these three zones. By maintaining the same position and orientation of the samples during µMRI, equal influence of collagen alignment on the *T*_2_ values was ensured for all samples. This influence, if present, can be expected to cancel out in comparisons between the samples.

Based on the relative contribution of BW and FW, to the observed 1/*T*_2_, the *T*_2_ evolution seen in the MSX joints (central coronal slice, Fig. 4 and Table 1) can be related to the physiological changes in AC during the study period of eight weeks. The minor positive *T*_2_ changes observed from week 1 to week 3 is interpreted as the excess FW due to the swelling initiated in week 1. Further *T*_2_ elevation was observed from week 4 to week 6, during which continued loss of PGs led to a further increase in FW and a simultaneous decrease in BW. This hypothesis is supported by the histology results that identified proteoglycan depletion and cellular loss at the week 4 time point (Fig. 2A). A rapid *T*_2_ increase of 37.6 ms was observed at week 7 as the result of severe proteoglycan loss, consequent decrease of the BW/FW ratio and increase in the free-water content. A pronounced inversion of the temporal trend of AC *T*_2_ was observed at week 8, when the *T*_2_ decreased by ~30 ms between weeks 7 and 8; this is attributed to the erosion of the cartilage and the accompanying loss of FW from AC. Nevertheless, a residual AC swelling appeared to remain because the *T*_2_ of the remaining AC was still longer than that of CTRL (Fig. 4). However, the AC *T*_2_ was observed to continually increase from week 1 to week 8 in the anterior and the posterior coronal slices (slice locations as shown in Fig. 1) and no decrease in *T*_2_ or AC erosion were observed (Table 2). It suggests that the progression of PTOA was more aggressive in the central (maximum load-bearing) coronal region than in the anterior or posterior regions of the joint.

### Cartilage Thickness and *T*_2_ as Biomarkers of PTOA

In this study, both the thickness and *T*_2_ of AC were observed to experience continuous changes during the progression of PTOA. *T*_2_ mapping has been established to be a sensitive marker of collagen content, distribution and orientation^47,56–59^ as well as of the proteoglycan content^60–62^, which influences the content of the free and bound water. Because the development of PTOA involves both proteoglycan depletion and collagen disruption prior to the erosion of AC, a marker sensitive to these changes would have definite advantages in identifying the physiological changes occurring in early PTOA. In a previous study, the correlation between the *T*_2_ values and the relative water content of AC was reported in a rat OA model at two time points^63^. In a partial-meniscectomy rat PTOA model, the AC of the weight bearing areas of the medial condyles exhibited a significant increase in *T*_2_ three weeks after surgery^64^. In another rat model, where PTOA was induced by the transection of ACL (ACLT), significantly higher *T*_2_ was observed in the AC of the operated knees at week 4 and week 13 post-surgery, the *T*_2_ value at week 13 was also significantly higher than that at week 4^63^. However, the results of these studies were insufficient to explain the relationship between PTOA and its effect on the *T*_2_ of AC due to the limited number of observational time points.

Our study has shown that, the thickness of AC and its *T*_2_ were strongly correlated (Spearman’s rank order correlation, *p* < 0.05) throughout the eight-week post-meniscectomy observation period, where week 8 marks advanced PTOA according to the Mankin score. However, there are also noticeable dissimilarities between the sensitivity of AC *T*_2_ and its thickness (Fig. 4) to the development of PTOA. The AC thickness in the MSX joints go through both positive (week 2, 3, 5 and 7) and negative (week 4 and 6) shifts, as seen in Fig. 4. These shifts were observed before the erosion of AC and therefore the reason for these apparent transient reductions in thickness is unclear. Additionally, both positive and negative shifts of the AC thickness were observed in the medial condyles of CLAT joints (Fig. 6) and in the lateral condyles of MSX joints (Fig. 7). The underlying reason for these changes in AC thickness cannot be explained within the scopes of this study. In contrast, the AC *T*_2_ continually increased from week 1 to week 7 (Fig. 4) and decreased with the commencement of cartilage erosion at week 8 in the medial condyle of MSX joints. Cartilage *T*_2_ remained steady in the medial condyles of CLAT joints (Fig. 6) and in the lateral condyles of MSX joints (Fig. 7). This dissimilarity can be attributed to the fact that AC thickness is a gross measure of the after-effect of the changes occurring in AC, while *T*_2_ provides insight into subtle compositional changes before AC erosion occurs. For example, the first significant change in *T*_2_ seen in the MSX joints (week 4) precede that in AC thickness (week 5) by one week. Similarly, the spike in *T*_2_ seen in week 7 precedes by one week the erosion of AC (week 8). The AC thickness measurement is also more severely affected by the resolution of the MR image in comparison to the cartilage *T*_2_ measurements. Therefore, *T*_2_ values of AC appear to be both an earlier and a more reliable indicator for understanding the course of PTOA than AC thickness.

### Changes in the Cortical and Trabecular Bone Volume of Epiphysis

Our results have identified significant decrease in epiphyseal *T*_2_ in the anterior, central and posterior coronal slices (slice location as shown in Fig. 1) of MSX and CLAT joints (Fig. 5), which were mutually correlated. The epiphyseal *T*_2_ represents the *T*_2_ of both subchondral bone and trabecular bone and therefore demonstrates the gradual reduction of water content within the tibial epiphysis from week 1 to week 8. Our visual observation of MR weighted images (TE = 24 ms) have identified two distinctive trends of subchondral bone remodelling: initial thinning followed by gradual thickening, between disease onset (week 1) and advanced PTOA (week 8).

The rats that underwent surgery had limited movement following surgery due to the surgical trauma and pain. Reduced movement of rats resulted in reduced amount of load on their tibial condyle. After the removal of medial meniscus, the joints had adopted to an alternative load distribution technique while the rats continued to move with functioning joints. According to our observations, this altered load distribution was the primary cause for the changed subchondral/trabecular bone volume ratio due to the bone remodelling within tibial epiphysis that resulted in gradual decrease in epiphyseal *T*_2_ from week 1 to week 8. According to the results presented in Fig. 5B, it can be stated with certainty that the epiphysis of the medial condyle experienced substantial alteration in all regions of the joint at each time point of observation.

In conventional practice, the quality of the subchondral bone is assessed by bone mineral density (BMD) and bone versus tissue volume ratio (BV/TV) measured over small cylindrical ROIs (few mm in diameter)^37,65^. BMD and BV/TV are computed from microcomputer-tomography (µCT) or X-ray scans of excised samples. In two PTOA rat models, where knee joints were subjected to ALCT alone or the combination of ACLT and MSX, damage to AC and subchondral bone loss was observed within 2 weeks of surgery^6^. This was followed by a significant increase in subchondral bone volume up to 10 weeks^6^. In a PTOA model of rabbit knee, bone loss or decreasing volume BMD were observed 4 and 8 weeks post-ACLT, and recovery to control values was observed at 12 weeks^66^. In a rabbit MSX model, initial changes of cartilage were associated with a decrease in BMD of the proximal tibia^65^. In a canine ACLT-MSX model, thinning and porosity of subchondral bone were observed in the medial condyles 12 weeks after the operation^5^. With the support of histological analysis and µCT data, thinning of subchondral bone was identified as a localised phenomenon related to cartilage degeneration, while trabecular bone changes were found to be related to mechanical loading^5^. In human post-mortem samples with early OA in proximal tibia, significant deterioration in the three dimensional architecture of cancellous bone and increased trabecular thickness and density with relatively decreased connectivity were observed, which suggested a mechanism of bone remodelling^38^. Due to the wide variability among the OA/PTOA models and the variable time points of measurements (that represent different developmental stages of PTOA), it is not possible to identify the exact time line of subchondral/trabecular bone remodelling in PTOA from the above mentioned studies. Additionally, due to the small field of view of µCT scanners, the sample must be excised before scanning, which is unsuitable for clinical practice or for monitoring the progression of PTOA. In contrast to the established CT-based protocol, we employed μMRI to measure the epiphyseal *T*_2_ for the assessment of subchondral bone and trabecular bone within epiphysis, which allows non-invasive evaluation.

### Effects of PTOA on Contralateral Joints

A very interesting finding of this study was the temporal variations of the epiphyseal *T*_2_ in medial tibial condyles of the contralateral joints. Without being subjected to any surgical procedure, the epiphyseal *T*_2_ of contralateral joints had significant deviation from controls and demonstrated striking resemblance to the joints subjected to meniscectomy with significant correlation (Spearman’s Rank Order Correlation analysis, *p* < 0.01). The epiphyseal *T*_2_ of the CLAT joints, in the central coronal slice, showed consistent reductions that continued up to week 8 (Fig. 5A). At week 8, the epiphyseal *T*_2_ of the CLAT joint reached 11.2 ms (see Table 1). This epiphyseal *T*_2_ is comparable to the epiphyseal *T*_2_ of the MSX joints between week 6 and week 7. This gradual reduction of epiphyseal *T*_2_ was identified in anterior and posterior coronal slices of the CLAT joints as well (see Table 2), yet, from week 1 to week 7, the epiphyseal *T*_2_ was higher in magnitude in the CLAT joints in comparison to the MSX joints.

In the studies concerning the development of experimental PTOA, contralateral joints are commonly ignored and considered to be unaffected. In fact, contralateral joints are also used as control data^7^. Figure 6 shows the comparisons between the following quantities of CLAT and CTRL joints: thickness of AC, *T*_2_ of AC and *T*_2_ of epiphysis. It is obvious that these properties of the normal or control (CTRL) are not similar to that of the contralateral (CLAT), particularly for the *T*_2_ of epiphysis. If the contralateral was taken as the control and if the effects of PTOA were determined by making comparisons, this study would likely result in a wrong understanding of PTOA development.

### Limitations of the Study

The resolution of the MR images acquired in this study was limited to the voxel size of 78 × 78 µm^2^. Although this choice of resolution allowed the *T*_2_ mapping of the intact limb containing the knee joint within a two-hour time slot, it has the potential to introduce a partial volume effect that could affect the quantitative accuracy of the MRI measurements. The AC of the rat joints was thin, and therefore it was not possible to differentiate one cartilage zone from another one at this resolution. The partial volume effect in the AC was corrected by mathematical processing based on Eqs (2) and (3) (see Materials and Methods). Nevertheless, a higher MRI spatial resolution would undoubtedly be beneficial to the quantitative accuracy of the measurements of the thickness and *T*_2_ of AC.

This study identified a gradual decrease of epiphyseal *T*_2_ in the medial epiphysis of MSX and CLAT joints that indicated a continuous remodelling of the subchondral and the trabecular bone within the medial epiphysis with the progression of the PTOA. However, the epiphyseal *T*_2_ could not unambiguously differentiate the *T*_2_ representing the subchondral bone from the *T*_2_ associated with the trabecular bone. Overall, the data acquired in this study did not reveal the exact nature of bone remodelling, or the factors underpinning the changes in epiphyseal *T*_2_. Further investigation is required, preferably involving both µMRI and µCT measures for a complete understanding of the epiphyseal bone remodelling in both MSX and CLAT joints.

### Feasibility of Monitoring PTOA Progression Using MRI

This study has shown that the three physical quantities: thickness of AC, its *T*_2_, and the epiphyseal *T*_2_, that are sensitive to the development of PTOA, can be measured from the *T*_2_-weighted images and the quantitative *T*_2_ maps. The use of the MSME imaging sequence allowed fast measurement of all three characteristics and minimised the time of sample exposure to room temperature (and therefore tissue degradation). The *T*_2_ maps were computed from a series of *T*_2_-weighted images acquired using the MSME sequence in the µMRI system. Partial volume effect, which often affects MRI measurements, was corrected by mathematical processing. Here, a single imaging modality (µMRI) was able to provide adequate information about the development of PTOA in the rat models. This marks a major methodological advance in the analysis of PTOA, in comparison with the standard practice, where a minimum of two diagnostic imaging modalities are used to assess the knee joint tissues: MRI for cartilage and CT for subchondral bone^66^. Due to the limitation of the bore size of the µMRI spectrometer used in this study, the limb containing the knee joint had to be removed and imaged on its own. Nevertheless, the knee was kept intact, including muscle and skin, and the limbs were maintained in osmotic conditions mimicking the physiological environment during the imaging. This augurs well for MRI-based comprehensive evaluation of PTOA *in vivo*, as our approach is in principle transferrable to clinical MRI scanners. At the same time, it must be kept in mind that imaging *in vivo* entails additional factors not present in sacrificed animals (most notably, motional and susceptibility artifacts resulting from the presence of active blood vessels), and the suitability of the present protocol for PTOA imaging *in vivo* ought to be demonstrated by further research.

Our imaging protocol and subsequent analysis identified the sequential changes in tibial cartilage and tibial epiphysis of rat knee joints by weekly observation for eight weeks following complete medial meniscectomy. Gradual swelling of AC was observed during the first week and continued for the next six weeks, while elevated water content resulted in an increase of the *T*_2_ values. Depletion of proteoglycan was identified in the fourth week that led to proteoglycan loss by the seventh week. Erosion of AC was observed in the eighth week, accompanied by a drop in *T*_2_ values. Although the thickness and *T*_2_ of AC were strongly correlated, *T*_2_ was clearly a more sensitive marker of the integrity of AC. The average *T*_2_ of epiphysis continued to decrease with the progression of PTOA. Integrating these observations, we identified the following disease development pathway that lead to advanced PTOA: meniscal injury → AC swelling (week 1–week 7) → bone remodelling in subchondral and trabecular region (week 2–week 8) → gradual depletion of proteoglycan and loss of cellular density (week 4–week 6) → severe proteoglycan loss and free-water influx (week 7) → erosion of the cartilage (week 8). Surprisingly, the contralateral joints also demonstrated altered epiphyseal *T*_2_, which evolved with time.

## Materials and Methods

### Rat OA Model

A total of 30 Male Wistar Kyoto rats (11–12 weeks old, 300–350 grams weight) were purchased from the Medical Engineering Research Facility (MERF, Brisbane, Australia) and housed in controlled day-night cycle (light/dark, 12/12 h) and controlled temperature (23 ± 1 °C). The 6 rats of the control group did not undergo surgery. PTOA was induced in the remaining 24 rats by complete medial meniscectomy (MSX) on the right hind knee joint^16,37^. The rats were anesthetised via intra-peritoneal injection with Zoletil (tiletamine 15 mg/kg, zolazepam 15 mg/kg) and Xylazil (xylazine 10 mg/kg), the medial collateral ligament was transected just below its attachment to the meniscus, the meniscus reflected towards the femur when the joint space opened and then the meniscus was cut at its narrowest point. This resulted in complete transection of the medial meniscus. Care was taken to avoid damaging the tibial surface. The surgical wound was closed by suturing the subcutaneous tissue and skin in two different layers. No surgery was carried out on the left hind knee.

The rats were allowed to walk freely in the cage after surgery. Pain killers (Buprenorphine 0.05 mg/kg) and antibiotics (Gentamycin 5 mg/kg) were given to the rats that underwent surgery. Following surgery, 3 rats were sacrificed every week (week 1–week 8) and 6 knee samples were harvested: 3 samples of the MSX joints and 3 samples of the CLAT joints. Three CTRL joints were harvested at week 1 and 3 were harvested at week 8. Each whole-joint knee sample extended from the middle of femoral diaphysis to the middle of tibial diaphysis (Fig. 1). The muscle and skin surrounding the knee joint were left intact in order to maintain an anatomically realistic environment. The samples were then subjected to MRI measurements. Animal ethics approval for this project was granted by the Queensland University of Technology (QUT) and the Prince Charles Hospital Ethics Committees (QUT Ethics approval number: 0900001134). All methods were carried out in accordance with the relevant guidelines and regulations of QUT.

### MRI Protocol

MR images were acquired at room temperature using a Bruker Avance NMR spectrometer (Bruker, Germany) at 7 T using 1.5 T m^−1^ (150 G cm^−1^) triple-axis gradient set, a Micro2.5 microimaging probe and a 25 mm radiofrequency (RF) birdcage ^1^H resonator coil. In order to maintain physiological osmotic conditions in the tissues imaged, the sample was hydrated for 2 hours in 0.01 M phosphate buffered saline (Sigma-Aldrich, USA) and then immersed in Fomblin (Sigma-Aldrich, USA) inside a 25 mm diameter NMR tube. The sample was positioned using purpose-built Teflon plugs^53,67,68^, with the axis of limb approximately parallel to the NMR tube axis and the static magnetic field (*B*_0_), which was maintained for all MRI scans.

The field of view (FOV) was determined by a 3D gradient-echo localiser scan using Fast Low-Angle SHot (FLASH) MRI sequence with repetition time (TR)/echo time (TE) of 100/5 ms, 2 mm slice thickness, 70 mm × 70 mm FOV, and 128 × 128 pixel matrix. Ten axial slices were acquired by multi-slice multi-echo (MSME) sequence with TR/TE of 1000/6 ms, 0.5 mm slice thickness, 30 mm × 30 mm FOV, 128 × 128 pixel matrix and 4 averages. Using these axial slices as references, 3 coronal slices were obtained by MSME with TR/TE of 5000/6 ms, 32 echoes, 0.5 mm slice thickness, 0.5 mm slice spacing, 20 mm × 20 mm FOV, 256 × 256 pixel matrix and 8 averages. The second coronal slice (as shown in Fig. 1) was positioned to contain the largest cross-section of ACL and PCL. The SNR was computed by taking the ratio of the mean pixel intensity in a region of interest (ROI) within the sample to the noise amplitude in a ROI of the background air (noise-only region). The noise amplitude was computed as the square root of the sum of the squared mean and the squared standard deviation of the signal in a noise-only region in a magnitude image (*μ*_*noise*_ and *σ*_*noise*_, respectively):

1 $$Noise=\sqrt{{{\rm{\mu }}}_{noise}^{2}+{{\rm{\sigma }}}_{noise}^{2}}$$

This measurement was repeated for ROIs in cartilage, muscle and tibial epiphysis. The SNR was maintained at a minimum of 9:1 for all tissues.

The week 4 CLAT joint samples were used to standardise the MR imaging protocol. The remaining 57 knee joints underwent the identical MRI data acquisition procedure discussed above. The data was divided into three groups: MSX group (24 right knee joints subjected to MSX: week 1–week 8), CLAT group (21 contralateral left knee joints: week 1–week 3 and week 5–week 8) and CTRL group (6 control right knee joints: week 1 and week 8).

### Histology

Soft tissues were removed from the joints after MRI. The joints were fixed in 4% paraformaldehyde, decalcified in 10% Ethylenediaminetetraacetic acid (EDTA), dehydrated and embedded in paraffin. A series of 5 µm coronal sections that matched the orientation and location of Slice 2 in Fig. 1 were then prepared from the medial tibial condyle of the joint. These sections were stained with Safranin-O/Fast Green^16,69^, which provided colour discrimination between bone and cartilage. The depth or thickness of AC was measured from histology stains using ImageJ (National Institutes of Health, USA) from the average distance (of three distance measurements) between the superficial borders of cartilage to the boundary with the calcified cartilage zone. The severity of PTOA was evaluated according to modified Mankin’s histologic grading system, ranked between 0–14, where 0 is the rank for normal and 14 is the rank for most severe OA^16,40^.

### MRI Measurement of Articular Cartilage Thickness

The thickness of AC was measured from the coronal MSME data sets at the relatively flat surface of medial tibial condyles, between the medial intercondylar tubercle of intercondylar eminence and the edge of the condyle. A straight line was drawn bordering the AC on the *T*_2_-weighted image (TE = 12 ms) as shown in Fig. 2 (line “a”, the yellow line). Ten lines perpendicular to line “a” were computed from femur to tibia (line “b”, the blue lines), the voxels nearest each line “b” were identified by rounding and a signal intensity profile was plotted along each line “b”. Three voxel intensities were specified: *I*_*C*_ for cartilage with the highest signal intensity, *I*_*F*_ for femoral cortical bone with no/minimal signal at the femoral end of cartilage and *I*_*T*_ for tibial cortical bone with no/minimal signal at the tibial end of cartilage. To correct for the partial volume effect, the volume fraction of articular cartilage, *P*_*AC*_ was computed using Eq. (2) for voxels located at the interface between the femoral cortical bone and cartilage, and using Eq. (3) for voxels at the interface between cartilage and the tibial cortical bone. *P*_*AC*_ was 1 for the voxels located entirely within cartilage. Partial volume correction was based on the assumption that the pixel with partial volume can only have two tissue types: cartilage and cortical bone of femur/tibia. Considering the signal intensity variation by slice position, *I*, *I*_*C*_, *I*_*T*_ and *I*_*F*_ were specified individually from the intensity profiles obtained from the *T*_2_-weighted image of each MRI slice.

2 $${P}_{AC}=\frac{I-{I}_{F}}{{I}_{C}-{I}_{F}}$$

3 $${P}_{AC}=\frac{I-{I}_{T}}{{I}_{C}-\,{I}_{T}}$$

Here, *I* is the signal intensity of a voxel at an interface between two tissue types.

The cartilage thickness was computed by multiplying the voxel dimension, 78 µm, by the sum of the *P*_*AC*_ measurements of each intensity profile. The mean of the 10 thickness measurements was taken as the AC thickness of the medial tibial condyle. The same procedure was followed to measure the AC thickness in the lateral condyle. The data analysis procedures, described in this section and in the following sections, were performed by in-house codes written in MATLAB R2014a (MathWorks, Natick, MA, USA).

### Measurement of Articular Cartilage *T*_2_

The quantitative *T*_2_ maps were computed from the coronal MSME data. The multi-echo data of every voxel was fitted with a three-parameter mono-exponential relaxation decay according to:

4 $$S={S}_{0}{e}^{\frac{-t}{{T}_{2}}}+{S}_{offset}$$

Here, *S* is the voxel signal intensity measured at the sequential MSME echoes, and *t* is the cumulative echo time (ranging in each data set through 32 equidistant values from TE to 32·TE). The fit parameters were *S*_0_ (full signal intensity), *T*_2_ (apparent spin relaxation time), and *S*_*offset*_ (the mean of the magnitude noise). With measured *S* and known *t*, the values of *T*_2_, *S*_0_ and *S*_*offset*_ were obtained by iterative least-squares fitting (LSF). A maximum of 100 LSF iterations were allowed for the voxels with *S*_0_ > 5 × *S*_*offset*_. All three LSF parameters were determined individually for every voxel. The number of voxels with *S*_0_ > 5 × *S*_*offset*_ varied between the imaging slices. However, in any given slice fewer than 3% of the voxels were identified as having *S*_0_ < 5 × *S*_*offset*_. The *S*NR was maintained at a minimum of 9:1 for all tissues in all slices. Fitting residuals were checked for randomness by Runs Test at α = 0.05 to verify the suitability of the mono-exponential fit given by Eq. (4).

For AC *T*_2_ of medial tibial condyle, voxels with *P*_*AC*_ > 0.5 were isolated from the voxel intensity profiles. The corresponding *T*_2_ values were then extracted from quantitative *T*_2_ maps using the voxel coordinates and the mean *T*_2_ was recorded. The voxels near intercondylar eminence and curved edges were excluded in order to avoid susceptibility artefacts. The same procedure was followed in the measurement of the cartilage *T*_2_ in the lateral condyle of the tibia.

### Measurement of *T*_2_ of Epiphysis

The mean *T*_2_ of tibial epiphysis was measured individually for the medial and lateral condyles. The coronal cross section of the tibial epiphysis is bordered by AC superiorly and by the growth plate or epiphyseal cartilage (EC) inferiorly (Fig. 1) where both AC and EC have much higher signal intensities compared to the cortical bone. Using the Sobel edge-detection filter on a *T*_2_-weighted image (TE = 24 ms), the medial compartment of the tibial epiphysis was outlined. A rectangular ROI was drawn at the centre enclosing 50% of epiphyseal area, two sides of epiphysis were excluded in order to avoid the chemical shift artefact. The corresponding *T*_2_ values were extracted from the quantitative *T*_2_ map using the voxel coordinates and the mean *T*_2_ was recorded. The same procedure was followed to measure the mean *T*_2_ in the lateral tibial epiphysis.

### Statistical analysis

The measurements of AC thickness, AC *T*_2_ and the *T*_2_ of epiphysis for the MSX, CTRL and CLAT groups over the 8-week observation period were entered in Matlab. For each physical quantity measured from each slice of each sample, a mean and a standard error was calculated every week from week 1 to week 8. This was done separately for the medial and the lateral condyles. The following nine data series (3 physical quantities × 3 groups of animals) were analysed for every slice location of medial condyle: AC thickness of MSX, AC thickness of CTRL, AC thickness of CLAT, AC *T*_2_ of MSX, AC *T*_2_ of CTRL, AC *T*_2_ of CLAT, epiphyseal *T*_2_ of MSX, epiphyseal *T*_2_ of CTRL and epiphyseal *T*_2_ of CLAT. The identical analysis was performed for the lateral condyle.

The Mann-Kendall trend test^70,71^ was performed individually on each of the thirty six data series (2 condyles x 3 slices x 3 physical quantities x MSX and CLAT group) to ascertain the presence of a temporal trend. For the data series of each slice (2 condyles x 3 slices), Spearman’s Rank Order Correlation analysis^72^ was performed to evaluate the correlation between the thickness of AC and its *T*_2_ values. In order to check for inter-slice correlations of epiphyseal *T*_2_ between MSX and CLAT joints and between the slices of the MSX joints, the Spearman’s Rank Order Correlation analysis^72^ was performed between the epiphyseal *T*_2_ data series of these groups at each slice location (2 condyles × 3 slices).
